# Eating Behaviour among University Students: Relationships with Age, Socioeconomic Status, Physical Activity, Body Mass Index, Waist-to-Height Ratio and Social Desirability

**DOI:** 10.3390/nu13103622

**Published:** 2021-10-16

**Authors:** Joanna Kowalkowska, Rui Poínhos

**Affiliations:** 1 Department of Human Nutrition, Faculty of Food Sciences, University of Warmia and Mazury in Olsztyn, Słoneczna 45F, 10-718 Olsztyn, Poland; 2 Faculty of Nutrition and Food Sciences, University of Porto, Rua do Campo Alegre, 823, 4150-180 Porto, Portugal; ruipoinhos@fcna.up.pt

**Keywords:** dietary restraint, uncontrolled eating, emotional eating, social desirability, body mass index, waist-to-height ratio, obesity, young adults

## Abstract

Eating behaviour is of particular interest for research focusing on body weight status. However, little is known about the relationships of certain factors, especially social desirability, with self-reported eating behaviour such as cognitive restraint, uncontrolled eating, and emotional eating among young adult males and females. This study aimed to evaluate the relationships between eating behaviour and age, socioeconomic status (SES), physical activity (PA), body mass index (BMI), waist-to-height ratio (WHtR), and social desirability among university students. A cross-sectional study was conducted among 353 university students (59.2% females). Eating behaviour was assessed using the 13-item Three-Factor Eating Questionnaire (TFEQ-13). SES and PA were determined using self-reporting, and the Marlowe–Crowne Social Desirability Scale assessed social desirability. BMI and WHtR were calculated based on measured parameters. Associations between self-reported eating behaviour and other variables were assessed using Pearson’s correlation coefficient and multivariate general linear models. Cognitive restraint was positively correlated with BMI and WHtR in both males (r = 0.174, *P* = 0.036 and r = 0.194, *P* = 0.020, respectively) and females (r = 0.239, *P* < 0.001 and r = 0.165, *P* = 0.017, respectively), and emotional eating was positively correlated with BMI among females (r = 0.184, *P* = 0.008). Social desirability was negatively correlated with uncontrolled eating (r = −0.287, *P* < 0.001) and emotional eating (r = −0.301, *P* < 0.001) among females. There were no significant correlations between eating behaviour and age or socioeconomic status (*P* > 0.05). Multivariate analysis showed that, among males, PA had a main effect on emotional eating (ηp^2^ = 0.044, F = 6.276, *P* = 0.013). Among females, cognitive restraint was positively associated with PA (ηp^2^ = 0.034, F = 7.127, *P* = 0.008) and BMI (ηp^2^ = 0.038, F = 7.959, *P* = 0.005), and emotional eating with BMI (ηp^2^ = 0.032, F = 6.638, *P* = 0.011). Social desirability had the highest main effect on eating behaviour among females, being negatively associated with uncontrolled eating (ηp^2^ = 0.077, F = 16.754, *P* < 0.001) and emotional eating (ηp^2^ = 0.082, F = 18.046, *P* < 0.001). This study showed that PA, BMI, WHtR, and social desirability were associated with self-reported eating behaviour among university students. Social desirability bias should be considered when evaluating uncontrolled eating and emotional eating among females.

## 1. Introduction

The prevalence of obesity almost tripled between 1975 and 2016 in the world [1]. In 2016, 39% of adults were overweight, and 13% were obese. The main cause of excessive body weight is an energy imbalance resulting from an increase in energy-dense food consumption and physical inactivity [1]. Eating behaviour dimensions are of particular interest in research focusing on the overweight [2,3]. 

Cognitive restraint (also referred to as dietary restraint) refers to strategies aiming to limit food consumption to maintain or reduce body weight [2]. However, it has also been associated with weight gain [4,5], as it can lead to increased hunger and appetite, resulting in an intense feeling of deprivation and the possible abandonment of dietary restrictions [5]. Restrained eaters may have problems with eating regulation, which can result in vulnerability to emotional eating and binge eating [4]. Uncontrolled eating and emotional eating are eating behaviour dimensions related to hunger and disinhibition [6]. Disinhibition refers to a tendency towards excessive eating, with usually hedonic food choices (i.e., energy-dense food, high in sugars and fat), potentially leading to poorer diet quality, obesity, and vulnerability to eating disorders [2]. Uncontrolled eating refers to the consumption of large amounts of food in response to food palatability, hunger, and social cues [2], while emotional eating refers to food consumption in response to stress or negative emotions, as physiological signals associated with emotions may be confused with hunger [5]. 

Eating behaviour is a complex construct influenced by a range of factors, including sex, age, body weight status, and psychological, social, economic, and lifestyle factors [3,7,8,9,10,11,12,13,14]. Previous studies conducted in European countries showed that levels of the eating behaviour dimensions differed by sex [15,16] and age [9,17]. Some studies showed that obesogenic behaviours (e.g., less healthy eating patterns, uncontrolled eating, lower physical activity) were more prevalent among people with lower socioeconomic status (SES) [7,10], but also that the association between SES and obesity risk was partly mediated by uncontrolled eating and eating at night [10]. Although cognitive restraint and emotional eating were higher among people with obesity, they were not significantly related to SES [10]. Regular physical activity (PA) can help to alleviate negative emotions [18] and improve appetite control [19], thus preventing overeating. PA can also compensate for excessive energy intake, thereby helping in weight loss or maintenance [19]. 

The assessment of eating behaviour and related factors based on self-report tools can be biased by social desirability [20], i.e., the tendency to avoid criticism and report more socially acceptable answers [21]. Social desirability bias may result in overestimation of healthy behaviours (or desirable traits) and underestimation of undesirable ones [22,23], and should therefore be considered [20]. Knowing the effect of social desirability on self-reported eating behaviour and its associations with other factors can be useful in improving the accuracy of dietary assessment and developing effective strategies to prevent eating and weight disorders.

Young adults starting independent life are particularly vulnerable to developing unhealthy eating behaviour, which can lead to eating disorders and/or becoming overweight. Some studies have explored the associations between such eating behaviour and several factors, including age [9,11,17], SES [9,10], PA [8,17], body mass index (BMI) and/or body weight status [3,9,11,24,25,26], and social desirability [16,27] among adults; however, very few have been conducted in Poland [25,26]. Some of the studies only included females [3,17,25,26]. Moreover, the relationships between some of the variables were inconsistent across studies. The discrepancies concerned, for example, the direction of the association between eating behaviour dimensions and SES [9,10] and between cognitive restraint and BMI, which may be population-dependent [9,10,24]. 

Therefore, the aims of the present study were to evaluate the association between eating behaviour dimensions (cognitive restraint, uncontrolled eating, emotional eating) among university students, their relationships with a range of factors (i.e., age, SES, PA, BMI, waist-to-height ratio (WHtR)), and to study the effect of social desirability on self-reported eating behaviour and on its relationships with the studied factors. 

## 2. Materials and Methods

### 2.1. Participants 

This cross-sectional study was conducted in a convenience sample of Polish university students. Students attending the University of Warmia and Mazury (Olsztyn, Poland) from different faculties and years of study were invited, in the university facilities, to participate in the study. The inclusion criterion was an age between 19 and 26 years. From a total of 365 participants, 12 were excluded due to the incompleteness of self-reported data. Thus, data from 353 students (59.2% females) were analysed. 

### 2.2. Procedure 

The study was conducted according to the guidelines of the Declaration of Helsinki and approved by the Bioethics Committee of the Faculty of Medical Sciences, University of Warmia and Mazury in Olsztyn on 17 June 2010 (resolution no. 20/2010); this approval was obtained for a period from 2010 to 2020 for a larger survey which was divided into several work packages. Data collection for the current study was conducted in the years 2014–2016 and continued in 2019 to obtain a larger sample size to perform the analysis for males and females separately. Potential participants were informed about the aims, scope, and organization of the study. Written informed consent was obtained from all participants. Respondents completed a paper-and-pencil self-administered questionnaire. For the current study, sections regarding eating behaviour, social desirability, sociodemographics, and lifestyle were considered. Anthropometric measurements were carried out by well-trained researchers at the university, according to the international guidelines [28,29]. The respondents’ height, weight, and waist circumference (WC) were measured using mobile stadiometers (SECA), digital scales (Tanita), and measuring tapes (SECA), respectively. Respondents were measured without shoes and in light clothing. Corrections for body weight and WC were applied (0.5 to 1.0 kg or cm, respectively). 

### 2.3. Measures

Eating behaviour was assessed using the Three-Factor Eating Questionnaire. The original version of the TFEQ was developed by Stunkard and Messick [30] and contained 51 items. The Polish version used in this study comprises 13 items (TFEQ-13) [31], and was developed based on the shorter, revised 18-item TFEQ developed by Karlsson et al. [6]. Twelve items were statements with four possible answers, indicating the degree of agreement with the statement and scored as follows: definitely yes (3 points), rather yes (2), rather not (1), or definitely not (0) [31]. The thirteenth item had an 8-point response scale, where 1 means ‘I never restrain from eating’ and 8 means ‘I always restrain from eating’ and was scored as follows: 1 or 2 (0 points), 3 or 4 (1), 5 or 6 (2), and 7 or 8 (3). The TFEQ-13 included three subscales: cognitive restraint (5 items; total score range: 0 to 15 points), uncontrolled eating (5 items; 0 to 15 points), and emotional eating (3 items; 0 to 9 points). The psychometric properties of the TFEQ-13 were evaluated among Polish teenagers and the variance explained by each of the three subscales was 28.9%, 19.2%, and 8.8%, respectively [31]. In the present study, good internal consistency was demonstrated for the TFEQ-13 subscales (Cronbach’s alpha was 0.803, 0.751, and 0.804, respectively). 

The socioeconomic status index (SESI) was created based on four categorical variables: mother’s education (three categories: primary/lower secondary, upper secondary, higher), father’s education (three categories: the same as for the mother’s education), family economic situation (three categories: below average, average, above average), and household’s economic situation (five categories: poor, modest, average, good, very good). Numerical values were assigned to each category in ascending order, e.g., mother’s education with three response categories: primary/lower secondary (1 point), upper secondary (2 points), higher (3 points). All variables were then standardized. SESI was calculated as the sum of the four standardized variables [7]. A holistic approach to determining the socioeconomic status of respondents (in one measure) was used in other studies [32,33,34], and the four-item SESI was previously used in a representative sample of 13–21-year-old Polish females [7]. In the present study, Cronbach’s alpha for SESI was 0.611. The SESI range in the study sample was from −10.25 to 6.49. SES index categories were based on a tertile distribution: low (35.1% of the sample), medium (30.9%), and high (34.0%). Details regarding the distributions of the SESI and its components in the total sample and by sex groups are shown in Table S1.

PA was assessed using questions regarding usual PA at work and/or school (“How would you describe your physical activity at work or at school?”) and PA at leisure time (“How would you describe your physical activity during your time off?”) [35]. Each question included three response categories (low, moderate, high). For each response category, a brief description with the amount of activity and examples of activities was given. Total PA was determined by a combination of certain categories of both questions, according to the categorization described elsewhere [35,36]. For example, total PA was considered ‘low’ when (i) low PA was reported by a respondent in both questions or (ii) low PA was reported in one of the questions, and moderate PA was reported in the other one. The reproducibility of the PA questions and total PA was previously assessed among adolescents and adults (15 to 65 years) [36]. Respondents were assigned to one of three categories of total PA: low (53.8% of the sample), moderate (42.8%), or high (3.4%). Due to the small number of participants with high PA, the ‘moderate’ and ‘high’ categories (46.2%) were combined for further analysis. Details regarding the distributions of PA at work/school, PA at leisure time, and the total PA in the total sample and within sex groups are shown in Table S1, and the participants’ characteristics by sex and PA subgroups are shown in Table S2. 

BMI as a measure of general adiposity [28] and WHtR as a measure of abdominal adiposity [37] were calculated based on measured height (cm), weight (kg) and WC (cm). BMI (kg/m^2^) was calculated as the body weight divided by the square of body height (in meters) [28]. WHtR was calculated as WC divided by the body height [37]. The distributions of BMI and WHtR as categorical variables in the total sample and within sex groups are shown in Table S1. 

Social desirability was evaluated using the Marlowe–Crowne Social Desirability Scale [21]. The scale contains 33 statements regarding personal attitudes and traits, with dichotomic answers (true/false), and with one point scored for answers corresponding to social desirability. The social desirability score was calculated as the sum of all items (range: 0 to 33). English to Polish translation (by two independent translators) and back-translation of the scale were performed. Cronbach’s alpha for this scale was 0.722. The test–retest reproducibility of the Polish version of the scale was assessed in the study sample (*n* = 353), showing a very good reproducibility of the social desirability score (r = 0.849, *P* < 0.001). A factor analysis was also performed in the study sample, and the scree-plot method showed that the scale presented a unifactorial structure (data not shown). 

### 2.4. Statistical Analysis 

Normality was tested using skewness and kurtosis. Since positive skewness was observed for WHtR, this variable was included in further analyses after logarithmic transformation. Descriptive statistics consisted of means and standard deviations (sd) for continuous variables and frequencies (n, %) for categorical variables. Means were compared using an independent sample *t*-test, and proportions were compared using chi^2^ or Fisher’s exact test. Since previous studies demonstrated sex differences in eating behaviour [9,15,16], further analyses were performed for males and females separately. Pearson’s correlation coefficient was used to measure the association between eating behaviour dimensions, age, SESI, BMI, WHtR, and social desirability. Partial correlations (controlled for social desirability) between eating behaviour dimensions and the other variables were also calculated. The effects of the studied characteristics on eating behaviour dimensions were assessed using multivariate general linear models (GLM), with effect sizes being measured with partial eta-squared (ηp^2^). All independent and dependent variables included in the multivariate GLM were continuous, except for one independent variable—the total PA (with two categories) [38]. 

The study sample size was tested using the post-hoc power analysis, based on mean scores obtained for eating behaviour among males and females. The statistical power was, on average, 83%, and ranged from 65% for uncontrolled eating to 93% for cognitive restraint. Overall, the post-hoc power was adequate (above 80%). 

All analyses were performed using PS IMAGO PRO 6.0 (Predictive Solutions, Cracow, Poland) and IBM SPSS Statistics for Windows, version 26.0 (IBM Corp., Armonk, NY, USA). *P* values < 0.05 were considered statistically significant. 

## 3. Results

Table 1 presents the characterization of participants regarding age, SESI, PA, BMI, WHtR, social desirability, and the TFEQ-13 subscales. All studied characteristics significantly differ between sexes, except for the social desirability. Age, SESI, BMI, and WHtR were higher among males. Moderate/high PA was found in 55.6% of males and 39.7% of females. Females scored higher on cognitive restraint and emotional eating, while males had higher uncontrolled eating. The characteristics of participants with low or moderate/high PA are available in Table S2. Similar sex differences in age, BMI, WHtR, and the TFEQ-13 subscales were found in both PA subgroups, except for emotional eating among adults with low PA and uncontrolled eating among those with moderate/high PA. 

The distribution of sociodemographic characteristics, physical activity, and body weight status in the total sample and by sex groups is shown in Table S1. Most of the sample had normal weight assessed with BMI (54.2% of males, 76.6% of females), and abdominal obesity was identified in 22.9% of males and 8.6% of females. 

Table 2 presents the correlations between eating behaviour dimensions, age, SES, BMI, WHtR, and social desirability among males and females. Regarding eating behaviour, in both sexes, the strongest correlations were found between emotional eating and uncontrolled eating. Emotional eating was positively correlated with cognitive restraint only among males. In both sexes, there was no significant correlation between eating behaviour and age or SES. BMI and WHtR were positively correlated with cognitive restraint in both sexes and BMI was positively correlated with emotional eating among females. Social desirability was associated with eating behaviour only among females, with negative correlations with uncontrolled eating and emotional eating. Partial correlations (controlled for social desirability) between eating behaviour dimensions and the other variables are shown in Table S3. These correlations did not differ significantly from those reported in Table 2 (*p* > 0.05 for all the comparisons), showing no significant effect of social desirability on the associations. 

Table 3 presents the effects of age, SES, PA, BMI, WHtR, and social desirability on eating behaviour. In the male subsample, only emotional eating was significantly explained by the independent variables, with low PA being related to higher emotional eating. Despite not being significant overall, WHtR was positively associated with emotional eating. Among females, all eating behaviour dimensions were significantly explained. Significant main effects were presented by PA, BMI, and social desirability. Participants with low PA had lower cognitive restraint, while higher BMI was associated with higher cognitive restraint and emotional eating. Higher social desirability was associated with lower uncontrolled eating and emotional eating. The parameter estimates for all significant effects have the same sign as the corresponding Pearson’s correlations. It is worth noting that the determination coefficients for each of the eating behaviour dimensions were higher for females than males, and in both subsamples they were higher for emotional eating.

## 4. Discussion

This study aimed to evaluate the associations of eating behaviour dimensions with age, SESI, PA, BMI, WHtR, and social desirability among university students. Based on the multivariate analysis, a main effect on emotional eating was presented by some of those variables in both sexes, while on cognitive restraint and uncontrolled eating among females only. PA, BMI, and social desirability presented a main effect on eating behaviour among females, with social desirability having the largest effect. PA was the only variable that presented a main effect on eating behaviour (i.e., emotional eating) among males. There was no significant association between eating behaviour and age or SES. In general, self-reported eating behaviour was better explained regarding emotional eating and among females. 

In the present study, cognitive restraint and emotional eating were higher among females, while uncontrolled eating was higher among males. Although females usually have higher body weight concern and can be more prone to limit food consumption to control their weight, they are also more vulnerable to eating in response to stress and negative emotions. The results suggest that men seem to be more prone to consuming large amounts of food, which may be due to food palatability, hunger, and social cues [2]. Similar sex differences in the scores of TFEQ subscales were found among French adolescents and adults, except for the scores of uncontrolled eating in middle-aged adults [15]. Higher scores of emotional eating and cognitive restraint among female students were also found in Portugal [16]. Research carried out among Lebanese and Chilean students also showed that females scored higher on emotional eating [11,39]. On the other hand, a study conducted among German adults showed that females scored higher on all three eating behaviour dimensions (TFEQ subscales) [9]. 

According to the current results, emotional eating and uncontrolled eating are positively correlated in both sexes, with a stronger association among females. Cognitive restraint and emotional eating had a low positive association among males only, and there were no significant associations between cognitive restraint and uncontrolled eating. Lower positive associations between emotional eating and uncontrolled eating were found among adult women and men in Germany [9], and between emotional eating and binge eating among students in Portugal [16]. Somewhat differently than in the present study, the association between cognitive restraint and emotional eating was significant but weak in both sexes among German adults [9]. Among Portuguese students, emotional eating was positively associated with different types of cognitive restraint: rigid control among females and flexible control among males [16]. Significant, positive associations were found between all TFEQ dimensions in post-menopausal women, with the strongest association between uncontrolled eating and emotional eating [23]. Although cognitive restraint can be considered both as a cause and a consequence of emotional eating [5], the cross-sectional study design did not allow a causal relationship to be assessed.

Interestingly, no significant association between self-reported eating behaviour and age or SES was found in the present study. This research was conducted among university students, and the participants’ SES characteristics were, to some extent, similar to other studies conducted among adolescents and young adults in Poland [7,33,40]. Some other studies demonstrated significant associations between some of the eating behaviour dimensions and age or SES. Age was positively associated with cognitive restraint and negatively associated with both disinhibition and hunger among female students satisfied with their weight [17]. Similarly, a positive association of age with cognitive restraint and negative associations with both uncontrolled eating and emotional eating were found among adult males and females in Germany [9]. Age was negatively correlated with uncontrolled eating only among male students in Lebanon [11]. The associations of SES with the three eating behaviour dimensions were positive but weak among adults in Germany [9]. In contrast, among adults in France, uncontrolled eating scores were higher among people with lower SES, whereas no significant relationships have been found with cognitive restraint or emotional eating [10]. Given the inconsistency in these findings, future research should include more objective SES measures and longitudinal studies. 

Positive correlations between cognitive restraint and both BMI and WHtR were found in both sexes, however, multivariate analysis confirmed a positive association between cognitive restraint and BMI only among females. Similar to the current findings, cognitive restraint has been positively associated with BMI among adult males and females in Germany [9]. In France, adults with obesity presented higher cognitive restraint [10], however, in Poland, no difference between women with normal weight and those with obesity was found [25]. The direction of the association between cognitive restraint and BMI may be different depending on the population studied: although those associations were weak, in a clinical sample of adults with obesity, cognitive restraint was inversely associated with BMI, while in a web-based survey among US adults, the association was positive [24]. Similarly, in the present study, further analysis showed significant positive correlations between cognitive restraint and BMI among non-overweight (BMI < 25 kg/m^2^) males and females, while they were negative (despite non-significant correlations) among overweight (BMI ≥ 25 kg/m^2^) males and females (data not shown). These findings suggest that adults with higher BMI demonstrated higher cognitive restraint only within a certain range of body weight status. On the other hand, cognitive restraint may have a counterproductive effect and lead to weight gain [4,5], since unsuccessful dieters can present high cognitive restraint and overeating tendencies [5]. Cognitive restraint has been indirectly associated with body size through its interaction with disinhibition [3]. 

The current results showed that emotional eating was positively associated with BMI among females. Among males, emotional eating was positively related to WHtR. Surprisingly, there was no significant association between uncontrolled eating and BMI or WHtR. Other studies have shown that both uncontrolled eating and emotional eating were related to BMI and/or body weight status [9,10,11,25]. BMI was positively associated with both uncontrolled eating and emotional eating among female students in Lebanon, while, among males, with emotional eating only [11]. Uncontrolled eating and emotional eating were higher among French adults with obesity (when compared to those without obesity) [10] and among Polish women with obesity (vs. normal weight) [25]. Other studies showed that dieting self-efficacy was negatively correlated with uncontrolled eating and emotional eating among Polish females aged 19–22 years [26], and eating self-efficacy with binge eating and emotional eating among Portuguese students (both males and females) [16]. Moreover, it was found that uncontrolled eating acts as a mediator in the relationship between SES and BMI among men [9]. Emotional eating did not mediate the association, but higher emotional eating scores were associated with higher BMI in both sexes [9]. One study showed that higher scores on both hunger and disinhibition were related to greater body weight and size among middle-aged and elderly women [3]. Interestingly, females with high disinhibition had higher BMI and WC compared to those with low disinhibition levels, irrespective of restraint level [3]. The current findings showed that higher emotional eating was associated with higher general adiposity among females and higher abdominal adiposity among males. This can be partly explained by physiological differences between both sexes—males have a higher tendency to accumulate abdominal visceral fat [41]. In the present study, abdominal obesity was found in about a 2.7 times higher proportion of males than females. Since PA plays a key role in the energy balance, the negative association between emotional eating and PA is relevant to the interpretation of such results. 

The current study also found that higher PA was associated with higher cognitive restraint among females and with lower emotional eating among males, although similar associations were expected in both sexes. Interestingly, among males, PA was the only variable that presented a main effect on eating behaviour. Only a few studies have explored the associations between TFEQ dimensions and PA [8,17]. Similar to the current findings, PA was positively associated with cognitive restraint and negatively with disinhibition among female university students dissatisfied with their weight [17]. People with higher BMI may restrain food consumption and/or increase PA to reduce body weight. The inverse association between PA and emotional eating may be explained by the role of PA in alleviating stress and negative emotions [18], and improving appetite control [19], therefore preventing overeating and weight gain. Another study, conducted among adult males and females, showed that emotional eaters with higher PA had lower BMI and consumed more healthy foods, even though there were no differences in the consumption of unhealthy foods (sweets, high-fat foods) [8]. Furthermore, self-reported PA may be biased by social desirability. In the present study, the social desirability score was higher among females reporting moderate/high PA compared to those reporting low PA (*p* = 0.024; data not shown). Other studies carried out among middle-aged women demonstrated that social desirability was associated with PA overreporting [22]. 

Contrary to expectations, no significant effect of the social desirability was found on the associations between eating behaviour dimensions and other variables. Nevertheless, social desirability presented a direct effect on some of the self-reported eating behaviours among females: uncontrolled eating and emotional eating were negatively associated with social desirability in both univariate and multivariate analyses. Social desirability had the largest main effect on eating behaviour, and it was the only factor which significantly explained uncontrolled eating. Among males, the associations with uncontrolled eating and emotional eating were also negative but non-significant. Overall, social desirability seems to be a potential bias when assessing eating behaviour among females, but not among males, or when regarding the relationships of eating behaviour with other characteristics. It is worth mentioning that there was no difference in mean social desirability scores between the sexes. Similarly, no sex differences were found in social desirability scores among Portuguese higher education students [16]. A relatively high mean social desirability score was found among post-menopausal women, which may be explained by actual positive traits of females who volunteered for research with a higher respondent burden [23]. As expected, females with higher social desirability scored lower on uncontrolled eating and emotional eating, which are eating behaviours considered socially undesirable and may be associated with feelings of shame or guilt [12]. The current findings are somewhat similar to those obtained among Portuguese students showing that social desirability was not associated with dietary restraint [16,27], but was negatively associated with external, emotional, and binge eating in both sexes [16]. A review of health-related studies demonstrated that socially desirable responding affected findings in almost half of those studies [20]. Only a few studies have explored the social desirability effect on associations between eating behaviour dimensions similar to those analysed in the present study [16,27], showing that controlling for social desirability weakened most of the associations [16]. 

### Strengths and Limitations

Several limitations of this study should be emphasized. Since the study was conducted among a sufficiently large, but convenience, sample of Polish university students, the findings may not be generalizable to other population groups and countries. Moreover, since this was a cross-sectional study, it is not possible to directly infer causal relationships. Difficulties in comparing the findings, to some extent, resulted from the different study populations (e.g., included females, middle-aged people, or subjects with obesity only) and tools (e.g., different TFEQ versions) applied in prior research. For example, the uncontrolled eating subscale in the TFEQ-R18 [6] included both hunger and disinhibition items of the original 51-item TFEQ [30], while the uncontrolled eating subscale in the TFEQ-13 comprised hunger items only [31]. Although this questionnaire was validated and used in other studies, it is a self-reported tool and does not take actual food consumption into account. 

The strengths of the study include using both univariate and multivariate analysis, allowing for the evaluation of the effect of each variable on eating behaviour adjusted for confounders, which strengthened the conclusions. Since a social desirability bias may affect the results based on self-reported data [20], partial correlations controlled for social desirability were calculated and showed no significant effect of social desirability on the associations between variables. Since some variables can be particularly sensitive to the social desirability bias, simple descriptive questions on the economic situation were used instead of a direct question about income. Anthropometric parameters were measured and two indexes reflecting general and abdominal adiposity (BMI and WHtR, respectively) were calculated. Besides using BMI, there were only a few studies on TFEQ dimensions, including other measures of body size such as WC [3,13], hip circumference, and waist–hip ratio [3]. 

The current findings highlight the most important factors that should be considered when assessing dietary restraint, uncontrolled eating, and emotional eating in future research, especially socially desirable responding. Besides the several factors included in the present study, based on the literature, some other factors related to the eating behaviour dimensions may be considered in future research: body weight satisfaction [17], nutritional knowledge [17], and night sleep duration [13]. To better understand the associations between eating behaviour dimensions and their causal factors, longitudinal research is needed. 

## 5. Conclusions

The current study showed that PA, BMI, and WHtR were associated with self-reported eating behaviour among university students. BMI was positively associated with cognitive restraint and emotional eating among females, while WHtR was positively associated with emotional eating among males. Among males with higher emotional eating, interventions aiming to prevent them from becoming overweight should focus on increasing PA. A social desirability bias was found when assessing self-reported eating behaviour, i.e., uncontrolled eating and emotional eating among females, but not on the relationships of eating behaviour dimensions with other characteristics. 

## Figures and Tables

**Table 1:** Participant characteristics.

	Total Sample	Males	Females	*P* Value
Total sample, n (%)	353 (100.0)	144 (40.8)	209 (59.2)	
Age (years), mean ± sd	21.3 ± 1.4	21.7 ± 1.6	21.0 ± 1.2	<0.001
SESI, mean ± sd	0.01 ± 2.71	0.37 ± 3.04	−0.24 ± 2.43	0.046
Physical activity, n (%)				0.005
low	190 (53.8)	64 (44.4)	126 (60.3)	
moderate/high	163 (46.2)	80 (55.6)	83 (39.7)	
BMI (kg/m^2^), mean ± sd	23.1 ± 3.9	24.9 ± 3.8	21.9 ± 3.5	<0.001
WHtR, mean ± sd	0.44 ± 0.06	0.47 ± 0.06	0.42 ± 0.05	<0.001
Social desirability, mean ± sd	15.5 ± 4.9	15.6 ± 4.4	15.4 ± 5.2	0.589
TFEQ-13 subscales, mean ± sd				
cognitive restraint	5.9 ± 3.3	5.2 ± 3.1	6.4 ± 3.4	0.001
uncontrolled eating	6.1 ± 3.1	6.6 ± 3.3	5.8 ± 2.9	0.011
emotional eating	2.8 ± 2.1	2.4 ± 1.9	3.1 ± 2.1	0.002

sd, standard deviation; SESI, socioeconomic status index, as a sum of four standardized variables (mother’s education, father’s education, family economic situation, household’s economic situation); BMI, body mass index; WHtR, waist-to-height ratio; TFEQ-13, 13-item Three-Factor Eating Questionnaire; *P*, significance level of the *t*-test for comparison between sexes.

**Table 2:** Correlations between eating behaviour dimensions, age, SESI, BMI, WHtR, and social desirability among males (*n* = 144) and females (*n* = 209).

	Cognitive Restraint	Uncontrolled Eating	Emotional Eating	Age (Years)	SESI	BMI (kg/m^2^)	WHtR	Social Desirability
	r (*P*)	r (*P*)	r (*P*)	r (*P*)	r (*P*)	r (*P*)	r (*P*)	r (*P*)
Cognitive restraint		−0.061 (0.467)	0.192 (0.021)	0.084 (0.317)	0.089 (0.286)	0.174 (0.036)	0.194 (0.020)	0.085 (0.312)
Uncontrolled eating	−0.097 (0.160)		0.428 (<0.001)	0.088 (0.295)	−0.080 (0.339)	0.133 (0.111)	0.125 (0.137)	−0.102 (0.224)
Emotional eating	0.112 (0.107)	0.601 (<0.001)		0.160 (0.055)	−0.101 (0.227)	0.052 (0.537)	0.155 (0.064)	−0.129 (0.124)
Age (years)	0.020 (0.768)	−0.134 (0.054)	−0.135 (0.051)		0.067 (0.428)	0.329 (<0.001)	0.318 (<0.001)	−0.033 (0.697)
SESI	−0.023 (0.745)	−0.029 (0.679)	0.063 (0.365)	−0.120 (0.084)		0.057 (0.500)	−0.021 (0.799)	0.196 (0.018)
BMI (kg/m^2^)	0.239 (<0.001)	0.074 (0.285)	0.184 (0.008)	0.047 (0.496)	−0.049 (0.480)		0.843 (<0.001)	0.009 (0.915)
WHtR	0.165 (0.017)	0.027 (0.697)	0.087 (0.209)	0.115 (0.098)	−0.073 (0.292)	0.858 (<0.001)		0.047 (0.573)
Social desirability	−0.049 (0.482)	−0.287 (<0.001)	−0.301 (<0.001)	0.024 (0.728)	0.057 (0.410)	−0.051 (0.460)	0.002 (0.977)	

Correlations for males are presented above the main diagonal, while correlations for females are presented below the main diagonal; r, Pearson’s correlation coefficient; SESI, socioeconomic status index; BMI, body mass index; WHtR, waist-to-height ratio.

**Table 3:** Effects of age, SESI, physical activity, BMI, WHtR, and social desirability on eating behaviour dimensions among males and females.

	Multivariate Tests			Tests of between-Subjects Effects											
				Cognitive Restraint (CR)				Uncontrolled Eating (UE)				Emotional Eating (EE)			
	F	*P*	ηp^2^	β	F	*P*	ηp^2^	β	F	*P*	ηp^2^	β	F	*P*	ηp^2^
Males (*n* = 144)															
Corrected model	-	-	-	-	1.337	0.245	0.055	-	1.302	0.260	0.054	-	3.314	0.004	0.127
Age (years)	0.990	0.400	0.022	0.037	0.046	0.831	0.000	0.104	0.331	0.566	0.002	0.175	2.966	0.087	0.021
SESI	0.629	0.598	0.014	0.081	0.835	0.362	0.006	−0.077	0.688	0.408	0.005	−0.039	0.556	0.457	0.004
Physical activity *	2.791	0.043	0.058	−0.457	0.745	0.390	0.005	0.908	2.650	0.106	0.019	0.784	6.276	0.013	0.044
BMI (kg/m^2^)	2.154	0.096	0.046	0.012	0.009	0.924	0.000	0.093	0.466	0.496	0.003	−0.144	3.500	0.064	0.025
WHtR	1.860	0.139	0.040	11.217	1.359	0.246	0.010	−0.012	0.000	0.999	0.000	12.013	4.457	0.037	0.032
Social desirability	0.793	0.500	0.017	0.038	0.386	0.536	0.003	−0.055	0.712	0.400	0.005	−0.045	1.548	0.216	0.011
Females (*n* = 209)															
Corrected model	-	-	-	-	3.587	0.002	0.096	-	3.918	0.001	0.104	-	6.027	<0.001	0.152
Age (years)	1.437	0.233	0.021	0.066	0.117	0.733	0.001	−0.307	3.606	0.059	0.018	−0.208	3.078	0.081	0.015
SESI	1.008	0.390	0.015	−0.028	0.092	0.763	0.000	−0.033	0.169	0.682	0.001	0.062	1.146	0.286	0.006
Physical activity ^*^	2.914	0.035	0.042	−1.267	7.127	0.008	0.034	−0.077	0.037	0.848	0.000	0.131	0.198	0.657	0.001
BMI (kg/m^2^)	4.526	0.004	0.064	0.356	7.959	0.005	0.038	0.093	0.769	0.382	0.004	0.201	6.638	0.011	0.032
WHtR	0.905	0.439	0.013	−8.641	0.831	0.363	0.004	−3.367	0.177	0.674	0.001	−8.290	1.995	0.159	0.010
Social desirability	7.737	<0.001	0.104	−0.037	0.717	0.398	0.004	−0.151	16.754	<0.001	0.077	−0.115	18.046	<0.001	0.082

* physical activity in two categories: low, moderate/high (as reference group); *P*, significance level; ηp^2^, partial eta-squared; β, standardized regression coefficient for parameter estimates; SESI, socioeconomic status index; BMI, body mass index; WHtR, waist-to-height ratio. Determination coefficients (R^2^) for eating behaviour dimensions among males: 5.5% for CR, 5.4% for UE, 12.7% for EE (adjusted: 1.4%, 1.3%, 8.9%, respectively); females: 9.6% for CR, 10.4% for UE, 15.2% for EE (adjusted: 6.9%, 7.8%, 12.7%, respectively).

## Data Availability

The data supporting the conclusions of this article will be made available from the corresponding author upon reasonable request.

## Supplementary Materials

The following are available online at https://www.mdpi.com/article/10.3390/nu13103622/s1, Table S1: Distribution of sociodemographic characteristics, physical activity, and body weight status in the total sample and within sex groups, Table S2: Participant characteristics by sex and physical activity subgroups, Table S3: Partial correlations (controlled for social desirability) between eating behaviour dimensions and age, SESI, BMI and WHtR among males and females.
