# Supplementary material for: Eating Behaviour among University Students: Relationships with Age, Socioeconomic Status, Physical Activity, Body Mass Index, Waist-to-Height Ratio and Social Desirability
Source: Nutrients. 2021 Oct 16;13(10):3622. doi: 10.3390/nu13103622 (PMC8541155; doi:10.3390/nu13103622)
Supplement: Supplementary file 1 [file nutrients-13-03622-s001.zip › nutrients-1355300-supplementary.pdf]

nutrients

MDPI

Eating behaviour among university students: Relationships with age, socioeconomic status, physical activity, body mass index, waist-to-height ratio and social desirability

Joanna Kowalkowska and Rui Poínhos

Supplementary Materials

Table S1. Distribution of sociodemographic characteristics, physical activity, and body weight status in the total sample and within sex groups.

	Total sample (n = 353)		Males (n = 144)	Females (n = 209)	*P* value
	n	%	%	%	
Place of residence	353	100.0	40.8	59.2	
village	130	36.8	41.0	34.0	0.018
town	123	34.8	38.9	32.1	
city *	100	28.3	20.1	34.0	
SES level					
low (T1)	124	35.1	33.3	36.4	0.013
medium (T2)	109	30.9	24.3	35.4	
high (T3)	120	34.0	42.4	28.2	
Mother's education					
primary/lower secondary	44	12.5	11.1	13.4	0.795
upper secondary	193	54.7	54.9	54.5	
higher	116	32.9	34.0	32.1	
Father's education					
primary/lower secondary	73	20.7	18.8	22.0	0.759
upper secondary	206	58.4	59.7	57.4	
higher	74	21.0	21.5	20.6	
Family economic situation					
below average	9	2.5	3.5	1.9	< 0.001
average	260	73.7	61.8	81.8	
above average	84	23.8	34.7	16.3	
Household's economic situation**					
poor	1	0.3	0.7	0.0	0.099
modest	5	1.4	2.1	1.0	
average	115	32.6	29.2	34.9	
good	206	58.4	56.9	59.3	
very good	26	7.4	11.1	4.8	

	**Total sample (n = 353)**		**Males (n = 144)**	**Females (n = 209)**	***P* value**
	**n**	**%**	**%**	**%**	
Physical activity at work/school					
low	185	52.4	43.1	58.9	< 0.001
moderate	139	39.4	41.7	37.8	
high	29	8.2	15.3	3.3	
Physical activity at leisure time					
low	151	42.8	34.0	48.8	0.021
moderate	114	32.3	38.2	28.2	
high	88	24.9	27.8	23.0	
Total physical activity					
low	190	53.8	44.4	60.3	< 0.001
moderate	151	42.8	47.9	39.2	
high	12	3.4	7.6	0.5	
BMI categories					
underweight (< 18.5 kg/m^2)	24	6.8	2.8	9.6	< 0.001
normal weight (18.5-24.9 kg/m^2)	238	67.4	54.2	76.6	
overweight (25.0-29.9 kg/m^2)	69	19.5	31.9	11.0	
obesity (≥ 30.0 kg/m^2)	22	6.2	11.1	2.9	
Abdominal obesity (WHtR ≥ 0.50)	51	14.4	22.9	8.6	< 0.001

*at least 100,000 inhabitants; SES, socioeconomic status (in tertiles); **household's economic situation – 5 categories (detailed description): poor (we live poorly – we do not have enough money for basic needs), modest (we live modestly – we have to be very careful with our daily budget), average (we live relatively thriftily – we have enough money for our daily needs, but we need to budget for bigger purchases), good (we live well – we have enough money for our needs without particular budgeting), very good (we live very well – we can afford some luxury); BMI, body mass index [28]; WHtR, waist-to-height ratio [37]; P, significance level of chi2 test (or Fisher's exact test) for comparison between sexes.

Table S2. Participant characteristics by sex and physical activity subgroups.

	**Total sample (n = 353)**	**Males (n = 144)**	**Females (n = 209)**	***P* value**
	**Mean ± sd**	**Mean ± sd**	**Mean ± sd**	
Low PA	(n = 190)	(n = 64)	(n = 126)	
Age (years)	21.3 ± 1.5	21.8 ± 1.7	21.1 ± 1.3	0.004
SESI	-0.16 ± 2.49	0.27 ± 3.05	-0.38 ± 2.13	0.131
BMI (kg/m^2)	23.4 ± 4.2	25.3 ± 4.3	22.4 ± 3.8	< 0.001
WHtR	0.45 ± 0.06	0.48 ± 0.07	0.43 ± 0.06	< 0.001
Social desirability	14.8 ± 5.0	15.1 ± 4.2	14.7 ± 5.3	0.586
TFEQ-13 subscales				
cognitive restraint	5.7 ± 3.0	5.0 ± 2.9	6.0 ± 3.1	0.034
uncontrolled eating	6.3 ± 3.1	7.2 ± 3.3	5.8 ± 2.9	0.004
emotional eating	3.1 ± 2.1	2.9 ± 2.0	3.2 ± 2.2	0.327
Moderate/high PA	(n = 163)	(n = 80)	(n = 83)	
Age (years)	21.3 ± 1.4	21.7 ± 1.6	21.0 ± 1.0	< 0.001
SESI	0.21 ± 2.94	0.46 ± 3.06	-0.02 ± 2.82	0.300
BMI (kg/m^2)	22.8 ± 3.5	24.6 ± 3.3	21.1 ± 2.9	< 0.001
WHtR	0.44 ± 0.05	0.46 ± 0.05	0.41 ± 0.03	< 0.001
Social desirability	16.2 ± 4.7	16.1 ± 4.4	16.4 ± 5.0	0.674
TFEQ-13 subscales				
cognitive restraint	6.2 ± 3.6	5.4 ± 3.3	6.9 ± 3.7	0.006
uncontrolled eating	5.9 ± 3.0	6.2 ± 3.2	5.6 ± 2.8	0.268
emotional eating	2.4 ± 1.9	2.0 ± 1.7	2.8 ± 2.1	0.005

sd, standard deviation; P, significance level of t-test for comparison between sexes; PA, total physical activity; SESI, socioeconomic status index, as a sum of four standardized variables (mother's education, father's education, family economic situation, household's economic situation); BMI, body mass index; WHtR, waist-to-height ratio; TFEQ-13, 13-item Three-Factor Eating Questionnaire.

Table S3. Partial correlations (controlled for social desirability) between eating behaviour dimensions and age, SESI, BMI and WHtR among males and females.

	Cognitive restraint	Uncontrolled eating	Emotional eating
	r (*P*)	r (*P*)	r (*P*)
Males (n = 144)			
Age (years)	0.087 (0.300)	0.085 (0.313)	0.157 (0.060)
SESI	0.074 (0.377)	-0.062 (0.464)	-0.078 (0.354)
BMI (kg/m^2)	0.174 (0.037)	0.135 (0.108)	0.053 (0.526)
WHtR	0.190 (0.023)	0.130 (0.121)	0.163 (0.052)
Females (n = 209)			
Age (years)	0.022 (0.756)	-0.132 (0.057)	-0.134 (0.054)
SESI	-0.020 (0.775)	-0.013 (0.853)	0.084 (0.226)
BMI (kg/m^2)	0.237 (< 0.001)	0.062 (0.372)	0.177 (0.011)
WHtR	0.165 (0.017)	0.029 (0.679)	0.092 (0.185)

SESI, socioeconomic status index, as a sum of four standardized variables (mother's education, father's education, family economic situation, household's economic situation); BMI, body mass index; WHtR, waist-to-height ratio.
